# Experimental phase determination of the structure factor from Kossel line profile

**DOI:** 10.1038/srep22904

**Published:** 2016-03-11

**Authors:** G. Faigel, G. Bortel, M. Tegze

**Affiliations:** 1 Wigner Research Centre for Physics, Institute for Solid State Physics and Optics, P.O.B. 49, Budapest, Hungary, H-1525

## Abstract

Kossel lines are formed when radiation from point x-ray sources inside a single crystal are diffracted by the crystal itself. In principle, Kossel line patterns contain full information on the crystalline structure: phase and magnitude of the structure factors. The phase is coded into the profile of the lines. Although this was known for a long time, experimental realization has not been presented. In this work we demonstrate experimentally that phases can be directly determined from the profile of the Kossel lines. These measurements are interesting not only theoretically, but they would facilitate structure solution of samples within extreme conditions, such as high pressure, high and low temperatures, high magnetic fields and extremely short times. The parallel measurement of many diffraction lines on a stationary sample will allow a more efficient use of the new generation of x-ray sources the X-ray free electron lasers (XFELs).

Traditionally, the atomic order in crystalline substances is determined by x-ray diffraction. Measuring diffraction peaks, we obtain the magnitude of the scattered waves, but the phase information is lost. However, for direct structure determination both the magnitude and the phase of the structure factors should be known. In practice, the loss of phase information is compensated by additional knowledge, such as atomicity, positivity of electron density, known part of the structure, composition, etc. Clever algorithms, like direct^1^, Patterson^2^, dual space^3^ methods, have been developed to incorporate and use our extra knowledge to solve the structure. Nevertheless, from time to time scientists tried to work out methods that allow experimental determination of the phase. The reason of this “quest” is that the knowledge of phase would greatly facilitate structure solution, especially in the case of non-centrosymmetric crystals. The best known approach is the controlled variation of the structure factor by various methods^4^,^5^,^6^,^7^. However, in this case special sample must be prepared, which limits applicability. Another approach is the three-beam diffraction, where three reflections are simultaneously excited^8^,^9^. Although it was shown that this method works, the practical application is not widespread, due to experimental difficulties. Another possibility is atomic resolution holography using inside sources or detectors^10^,^11^,^12^. This method can give the local atomic arrangement in orientationally ordered samples by a direct transformation of the holographic pattern which contains the phase. However, the measurement of x-ray holograms are technically complicated, time consuming and only relatively simple structures (10–100 atoms) can be determined.

Hutton, Trammell and Hannon^13^ and in an independent work Stephan *et al.*^14^ suggested the use of Kossel line patterns as source of phase information of diffraction peaks produced by inside x-ray sources. Hutton and co-workers based their method on the analysis of the fine structure of individual Kossel lines. They gave a theoretical prediction for the angular dependence of the Kossel line profile. They showed that in the Bragg case and in special conditions^13^ the line shape strongly depends on the phase of the structure factor (see Supplementary Movie 1). Although they described the necessary experimental conditions in detail, there has been no experimental work measuring a Kossel pattern (i.e. many Kossel lines in parallel) and determine the fine structure of all lines, so far. There has been a pioneering experiment^15^, in which the fine structure of a single Kossel line was measured, and its phase was determined, within very special conditions. The reason, why these type of measurements are scarce is the technical difficulties (see Supplementary Information, “Design Considerations of the Experimental Setup” and Ref. 16). In this paper we present experimental evidence for the direct determination of the phase of structure factors from the fine structure of measured Kossel lines. Further, we show that the special conditions given in Refs 13,15 can be relaxed, leading to wider applicability. We also discuss the unique possibilities given by the Kossel technique, i.e. obtaining structural data on a steady sample at extreme conditions such as very high pressures, temperatures or magnetic fields within a very short period of time. With these extensions this method will allow to solve presently intractable problems in diverse areas of natural sciences.

## Theory

**Figure 1:**
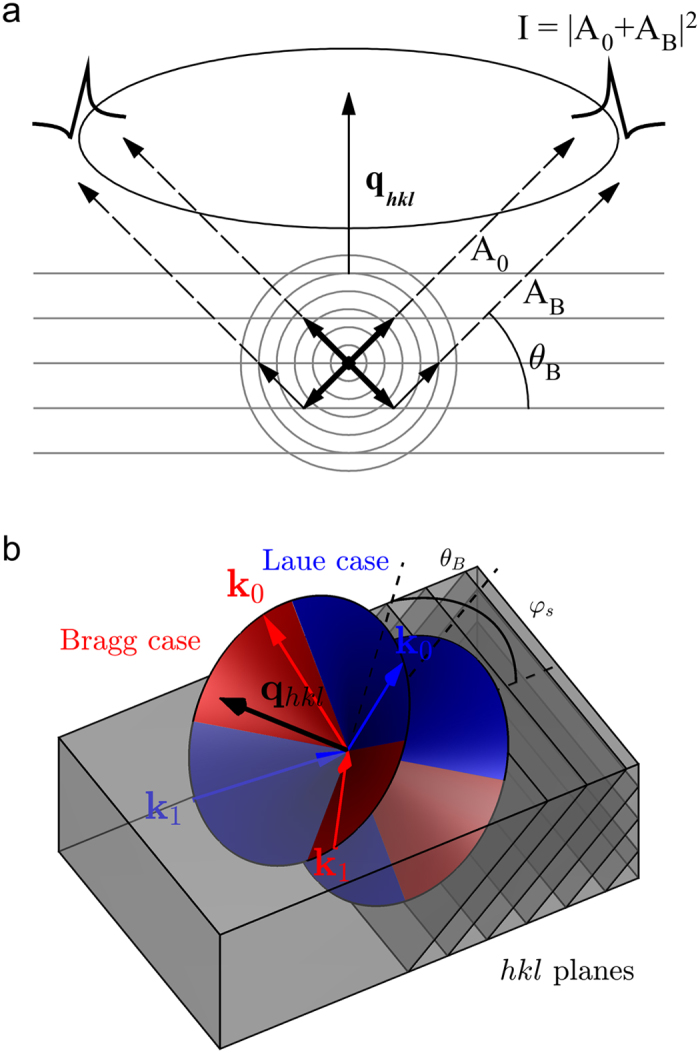
(**a**) Formation of Kossel lines. Fluorescent radiation emitted by the atoms (A_0_) in the sample interferes with its Bragg reflection (A_B_) resulting in strong intensity modulations (I = |A_0_ + A_B_|^2^) in the vicinity of cones defined by the normal of the diffracting plane (*hkl*) and the Bragg angle (*θ*_B_). The viewing direction is in the diffracting planes (grey lines) but the cone is illustrated with an ellipse. Arrows indicate relevant direct (thick arrows) and reflected (thin arrows) plane-wave components of the spherical-wave fluorescent radiation. (**b**) Bragg and Laue case of Kossel lines. **k**_0_ and **k**_1_ wave vectors (see text for definition) are located on the same side of crystal surface for the Bragg case (red), while on the opposite sides for Laue case (blue). There exist pure Bragg, pure Laue and mixed Bragg-Laue cones (shown in the figure) depending on *θ*_B_ and *φ*_s_ (the angle between **q**_*hkl*_ and the crystal surface normal). For more details see Supplementary Movie 2.

Kossel lines are formed when atoms of a single crystal emit x-ray photons and these are scattered by the crystal itself. For easy visualization of this process, we depicted schematically the formation of Kossel lines in Fig. 1a. The regions of the Kossel cone corresponding to the “Laue case” (often called transmission geometry) and “Bragg case” (often called reflection geometry) in the terminology of dynamical diffraction are explained with the help of the crystal surface, the crystallographic layers and the beam directions (Fig. 1b). Since these geometries are important in the evaluation process of our experiment, we refer to this figure later. We point out here that the geometrical difference between the two cases leads to a significant difference in the phase change of the diffracted wave, going through the reflection region. The phase of the diffracted wave changes π in the Bragg case while much less in the Laue case (about twice the phase angle connected to the anomalous correction). This shows up in the different angular dependence of the Bragg and Laue case Kossel lines profiles (see later equations (1) and (2)). The formation of Kossel lines was experimentally shown by Kossel, Loeck and Voges^17^ and theoretical description was given by Laue^18^. Detailed theoretical description of the fine structure of these lines was given by Hutton, Trammell and Hannon^13^. Applying the theory of dynamical diffraction for the inside source case for thick single crystals in which the position of fluorescent atoms are restricted close to the top surface of the sample (close means less than the extinction length), they concluded that the angular dependence of the intensity crossing a Kossel line is characteristic to the phase of the structure factor involved (see Supplementary Movie 1). Their description was given for the Bragg case lines (see Fig. 1 and Supplementary Movie 2). In practice, the above restrictions severely limit the usability of the approach. First, one needs a special sample or a special geometry to have emitters only in the top surface. Secondly, Bragg case lines in our geometry are usually weak, because one needs high Bragg angles to satisfy the Bragg case condition. The most intense Kossel lines are most often produced in the Laue case (see Fig. 1 and Supplementary Movie 2). However, in the Laue case no simple closed analytical form exists for the description of the fine structure of Kossel lines. A more elaborate treatment of the geometry of the experiment is needed. Therefore we turned to earlier works on the inside source problem developed for nuclear sources and scatterers^19^,^20^,^21^. The theory worked out in these papers included the more general case of polarization mixing by the scattering medium and higher multipolarity inside sources. Since in our case the sources are isotropic E1 emitters and the scattering is by the electrons (i.e. electric dipole), we used a simplified form of the expressions given in these papers. For simplicity, we omitted polarization, which has negligible effects. Further, we applied the formulas for the general case when the full sample volume contains emitters. We resorted to numerical solutions both in the Laue and in the Bragg case. With these modifications, our measurements could be analyzed and the phases for the measured reflections could be obtained. We give here only the final formulas used in the evaluation (for derivation of these formulas see Refs 19, 20, 21 and the Supplementary Information, “Derivation of Equations (1) and (2)”). Our notation mostly follows the convention of^19^,^20^,^21^ for easier understanding.

The contribution of a source atom located at **r**_*j*_ in the *M*_*j*_-th layer to the reflection *hkl*, at a given wavelength *λ* is given by 2 separate formulas for the Bragg and the Laue cases.

For the Laue case:


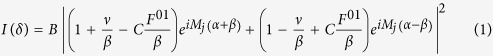


where,


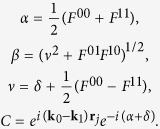


For the Bragg case:


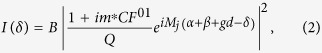


where


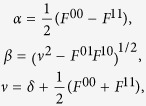



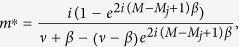



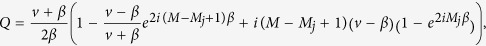



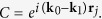


The following variables are the same for the two cases:


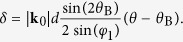


*δ* is related to the angular deviation from the kinematical Bragg angle *θ*_B_; *M*_*j*_ is the serial number of the layer containing the source atom below the upper surface of the crystal, *M* is the total number of layers, **k**_0_ is the direct wave wave-vector, **k**_1_ is the wave-vector of the wave which is scattered into **k**_0_ satisfying the Bragg condition (**k**_0_ = **k**_1_ + **q**_hkl_ where **q**_hkl_ is the reciprocal lattice vector of the reflection under consideration and the scattering is elastic; |**k**_0_**| = **|**k**_1_**| = **2π/λ), *g* is the projection of **k**_0_ to the normal of the crystal surface, **r**_*j*_ is the position of the source atom relative to the origin of the unit cell, *B* is a normalization factor, which describes the intensity of the Kossel line relative to the total off-Bragg intensity and can be calculated from the fluorescent yields. *θ*_B_ is the Bragg angle, *θ* is the rocking angle, *φ*_1_ (*φ*_0_) is the angle between **k**_1_ (**k**_0_) and the crystal surface, *d* is the interplanar spacing, *F*^00^, *F*^11^, *F*^01^ and *F*^10^ are the planar scattering amplitudes (for definition see equations (7) and (8) in Ref. 20).

The total intensity variation is calculated by summing the contributions of all layers containing source atoms. Since our goal is to determine the phase (*Φ*) of the structure factor we invoke here the dependence of *F*^01^ on *Φ*: *F*^01^ = *R* e^i*Φ*^ where *R* is a real constant^20^. Note, that in traditional crystallography the origin choice of the unit cell affects the phase of reflections via a phase-factor. In our measurement the source atom fixes the origin and through this the phases of the Kossel lines. When comparing the phases obtained from our measurements (*Φ*_m_) and the phases calculated from the known structure of GaAs (*Φ*_c_), we placed the origin to the Ga or As sites according to the experimental conditions, and corrected the measured values by the corresponding phase factors.

## Experiment

**Figure 2:**
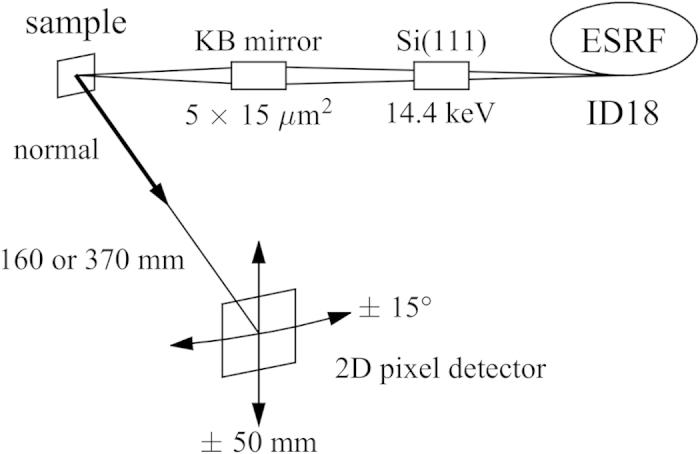
Experimental setup. A focused monochromatic synchrotron x-ray beam was used to excite atoms in the sample. The emitted fluorescent radiation (modulated by the interference between the direct and Bragg reflected beams) were recorded with a 2D pixel detector. To increase the solid angle covered by the measurement, the detector assembly can be moved.

**Figure 3:**
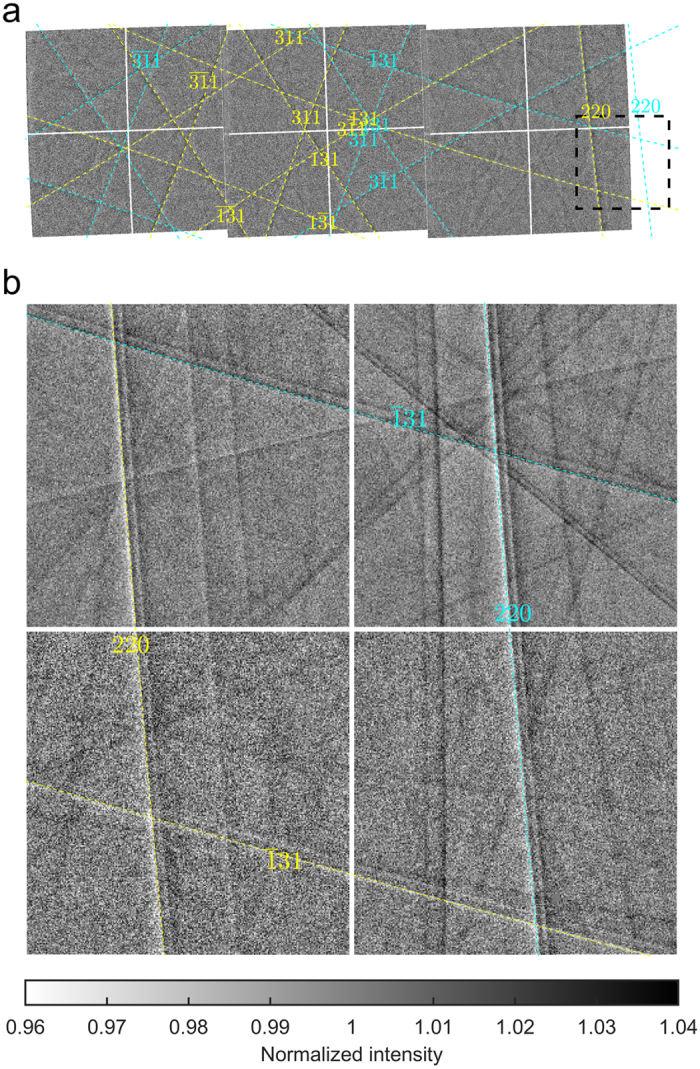
Measured Kossel patterns. (**a**) 3 aligned images taken with short detector distance (160 mm) were used for indexing the lines and selecting a region (black dashed line box) for the measurement of the line profiles. (**b**) The image taken in the selected region with larger detector distance (370 mm) was used to extract the Kossel-line profiles. Cyan and yellow colors indicate strong Ga K_α1_ and As K_α1_ lines. Other energies and reflections are not shown for better visibility of the raw pattern.

The Kossel patterns were taken at ESRF at the ID18 beamline. We have built a special setup to collect the patterns. The details of the setup are described in Ref. 16 and the Supplementary Information, “Experimental Setup”. Here we give those parameters, which are special to the present experiment only. First, the choice of sample material: for a demonstration experiment the best sample is a relatively simple, well known crystal. However, at the same time, it should have structure factors with non-trivial phases, so that many different phases could be obtained, reflecting the strength of the method. Finally, a practical condition is the crystal quality, which should be good enough not to broaden too much the diffraction lines allowing the line shape analysis. All these requirements are satisfied by a GaAs single crystal. GaAs has a cubic zinc blende structure with space group 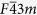. This structure consists of two interpenetrating face-centered cubic lattices, one for Ga and one for As. There are single non-equivalent sites for Ga and As in the unit cell. This simplifies the evaluation procedure, since having more non-equivalent source atoms in the unit cell would require the determination of the relative position of these atoms before the phase analysis. The choice of GaAs as a test sample gives a further advantage, the possibility of simultaneously measuring both the Ga and the As Kossel lines, since the beamline (ID18) is optimized for the 14.4 keV radiation, which is just above the absorption edges of both elements. Since well-separated emission energies give separated Kossel profiles, this allows a multiple check of the results. The geometry of the experiment is shown in Fig. 2. Since the size of our detector was small (25 × 25 mm^2^ active area, with pixel size 50 × 50 μm^2^) we carried out two sets of measurements: one with the detector close to the sample (160 mm) and another one at a larger detector distance (370 mm). The first set covered larger solid angles by combining 3 adjacent images, showing more lines but with moderate angular resolution (Fig. 3a). This pattern is used to find the orientation and index the lines. The second set (Fig. 3b) – with higher angular resolution – provides the angular profiles of some selected lines. We chose this area to include lines having different phases. Figure 3 shows the Kossel line pattern after removing the general fluorescent background, and correcting for the detector segment and pixel inhomogeneity. Detailed description of the pre-processing of the measured patterns is given in Ref. 20 and the Supplementary Information, “Pattern Processing”.

## Result

We could identify 26 Kossel lines; 14 of these were produced by Ga and 12 by As source atoms. From the 14 Ga lines 10 were Ga K_α1_ and K_α2_ flourescence, while 4 were Ga K_β_ fluorescence. From the 12 As lines 9 were As K_α1_ and K_α2_ flourescence, while 3 were As K_β_ fluorescence. The angular distance of K_α1_ and K_α2_ lines is comparable to the broadened width of the Kossel profile requiring their simultaneous description, while the K_β_ lines are well separated and can be treated separately. The Kossel line profiles for each reflection and emission energies were extracted by averaging measured intensities along narrow concentric cones of the given Kossel cone. This is a non-trivial procedure, because for non-symmetric reflections (i.e. the axis of the cone is not perpendicular to the surface of the crystal) the profile parameters depend on the position where we cross the cone, or even it can change the case from Bragg to Laue or vice-versa. (see Fig. 1 and Supplementary Movie 2). However, this dependence is a slowly varying function of the azimuthal angle; therefore we could take the profile constant in the small angular range covered by our detector. Sorting the lines by cases they correspond to 2 Bragg and 24 Laue case lines. Profiles were fitted to these lines according to equations (1) and (2). In principle, there are four parameters to fit: *F*^01^, *F*^10^, *F*^00^ and *F*^11^. Examining equations (1) and (2) we see that *F*^00^ and *F*^11^ forward scattering amplitudes determine the shift of the diffraction lines relative to the kinematical Bragg angle. Their absolute value is proportional to the number of electrons in the unit cell, and their imaginary part is determined by the absorption. These values are known, given by the sample composition^22^. In general, their values are small; the shifts are in the arcsec range. Since we cannot measure absolute angular positions with this precision by our setup, there is a systematic error in the peak positions determined by the uncertainty in the geometry of the detector-sample assembly. Therefore we have to fit the position of the peaks, in spite of knowing *F*^00^ and *F*^11^. However, this way the fitted values of the peak positions cannot be used as a consistency check for *F *^00^ and *F*^11^. In what follows we do not draw any conclusions from the fitted position parameters. For phase determination, the important parameters are *F*^01^ and *F*^10^. Their phase determines the shape of the Kossel lines. By definition, *F*^01^ and *F*^10^ are not independent; *F*^01^ = *A*_0_/sin(*φ*_1_) × e^i*Φ*^ while *F*^10^ = *A*_0_/sin(*φ*_0_) × e^−i*Φ*^ where *A*_0_ is the magnitude of the structure factor and *Φ* is its phase. This means that we have to fit the Kossel line profile by a minimum of 3 parameters: *A*_0,_ *Φ* and a position parameter. However, in practice we have one more fitting parameter determined by the experimental conditions: the line broadening. It comes from three factors: the energy width of the fluorescent lines, the imperfection of the crystal, and the angular resolution of the experimental setup. The energy width of the fluorescent lines can be precisely given but the other two factors change from line to line depending on the geometry and in practice they cannot be easily derived. Estimating their contributions we found that the crystal imperfection dominates, followed by the angular resolution of the setup, and the smallest contribution comes from the energy width of the fluorescent lines. We took into account the line broadening by convoluting the theoretical lines (given by equations (1) and (2)) with a Gaussian. In the fitting procedure we obtained 20–100 times total broadening of the theoretical line width. It is important to point out here, that the line broadening and *A*_0_ are correlated in the fitting procedure. Therefore no conclusive information can be drawn for any one of them. However, this does not affect the determination of *Φ*, which is our goal. We show below, that even with this relatively large broadening the line profiles give the phase with about 10° precision, which is less than 3 percent of the range of possible phases.

**Figure 4:**
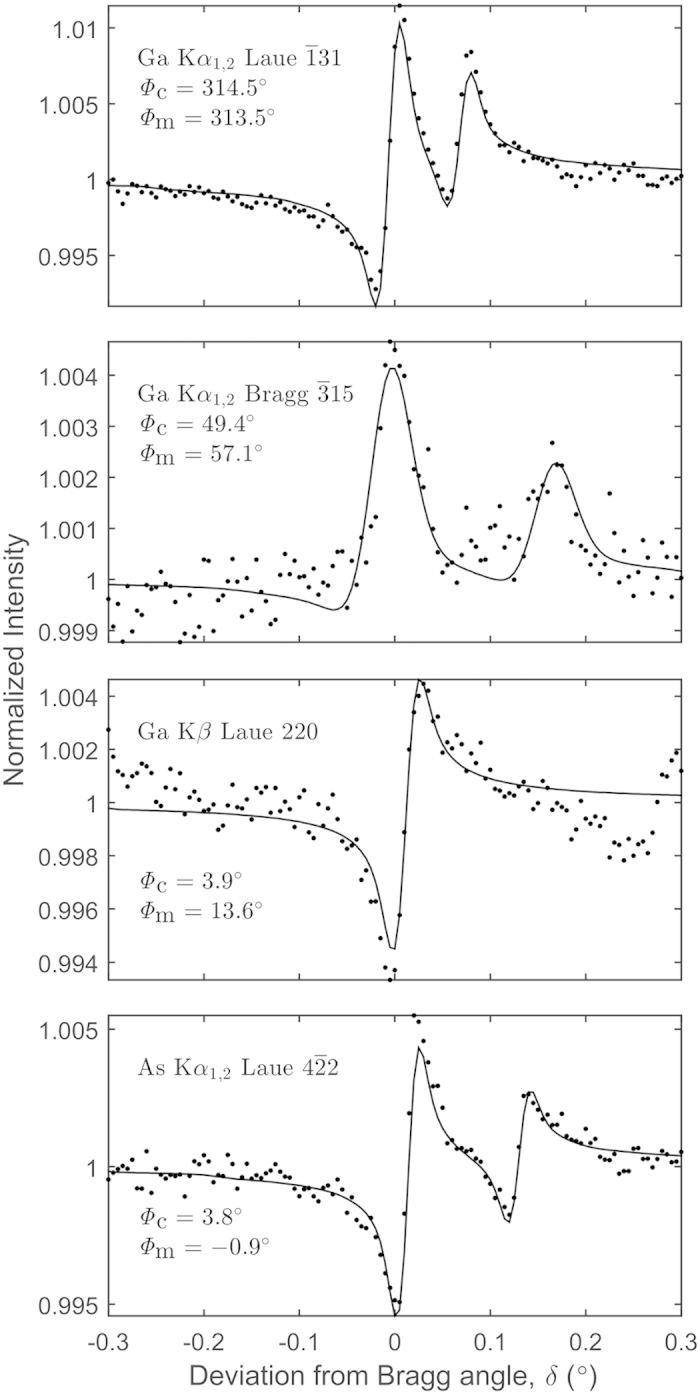
Measured and calculated Kossel-line profiles (dots and solid lines) of 4 selected reflections and source energies. *Φ*_c_ and *Φ*_m_ are the calculated and the measured phases of the structure factors. The intensity is normalized by the off-Bragg fluorescent background, resulting in a baseline of 1.

In the fitting procedure we fitted 4 parameters: line position, line broadening, amplitude of structure factor and phase of the structure factor. The procedure was a 3-stage process: first we found approximate starting parameters for the iteration. This was done by choosing estimated values for the position, broadening and magnitude of the lines, meanwhile mapping the phase (*Φ*) from 0–360° in 20° steps. We selected the starting phase to the value where the line shape was closest to the measured one. In the second stage we fixed the phase and performed a 3 parameter nonlinear minimization of the sum of squared differences of the measured and calculated profiles. This gave the experimental parameters: the width of the Gaussian, the positions and amplitudes of the lines. In the third stage, we fixed the experimental parameters and iterated for the phase. We numerically checked the convergence region and uniqueness of the fitted parameters and found that the above process is unbiased (see Supplementary Material, “Line-shape, Fitting, Convergence and Uniqueness”). In Fig. 4 four typical profiles are shown as illustration. We selected these to include various types of lines and different phases. The fits are reasonably good. The other lines are similarly well described, and the phases agree within a few degrees with the theoretical values (calculated from the GaAs structure, taking into account the anomalous corrections, too). The theoretical (*Φ*_c_) and the experimentally (*Φ*_m_) determined phases are given in Table 1. In two cases we have somewhat larger than 10° error (220 and 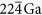 lines). We attribute these larger errors to the non-perfect deconvolution of the background contributions. It should be mentioned here that the error of the phase found at other lines is also dominated by the same type of error and not by statistical error. More precise phases could be obtained by using better detectors and a stricter control of the background caused by the scattering of the incident beam and the fluorescent radiation by the environment (goniometer parts, shielding, air, etc., see a more detailed discussion in the Supplementary Material, “Experimental Considerations in XFEL Measurements”). We hope that in the near future these technical problems will be solved and this method reaches its full power.

## Conclusion

In this work we illustrated the possibility of retrieving quantitative phase information from Kossel line patterns. These results show that parallel phase determination of several structure factors can be experimentally realized. Using the proper evaluation tools, phases of 26 reflections of a GaAs crystal were obtained. Beside the theoretical interest, this measurement opens the way to several applications. One of the most important advantages of this experimental arrangement is that a single crystal pattern (many Bragg reflections) can be taken at a single position of the sample simultaneously. This allows detailed structural characterization of changes caused by highly non-ambient conditions such as high magnetic fields, very high pressures, temperatures etc. Further, setting up this experiment at free electron laser sources, one could get a full diffraction pattern from a single pulse. This allows the characterization of very short-lived structural features, in the 10–100 fs range, even for irreversible processes. All these are applicable if we use only the geometrical information i.e. the position of the Kossel lines. If we add the extra phase information, this method can revolutionize structure determination at extreme conditions.

## Additional Information

**How to cite this article**: Faigel, G. *et al.* Experimental phase determination of the structure factor from Kossel line profile. *Sci. Rep.* **6**, 22904; doi: 10.1038/srep22904 (2016).

## Supplementary Material

Supplementary Information

Supplementary Movie 1

Supplementary Movie 2

## Figures and Tables

**Table 1:** The reflection table lists the crystallographic indices (*hkl*), the calculated phase of the structure factor (*Φ*_c_), the measured phase obtained from the Kossel profile (*Φ*_m_), their difference, the Laue/Bragg case and the source of the Kossel line.

*h k l*	Φc (°)	*Φ*_m_ (°)	*Φ*_c_–*Φ*_m_ (°)	L/B	source
3 1 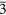	320.3	313.1	7.2	L	As Kβ
2 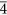 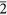	3.2	5.6	−2.4	L	As Kβ
0 4 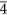	3.2	−6.5	9.7	L	As Kβ
2 2 0	3.9	13.8	−9.9	L	As Kα_1,2_
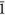 3 1	47.2	47.5	−0.3	L	As Kα_1,2_
1 3 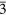	46.6	36.3	10.3	L	As Kα_1,2_
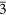 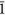 3	46.6	37	9.6	L	As Kα_1,2_
4 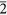 2	3.8	−0.9	4.7	L	As Kα_1,2_
1 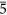 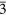	321.6	315.5	6.1	L	As Kα_1,2_
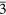 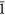 5	321.6	318.5	3.1	L	As Kα_1,2_
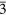 3 5	321.8	311.9	9.9	L	As Kα_1,2_
3 5 3	45.4	39.5	5.9	L	As Kα_1,2_
2 2 0	3.9	13.6	−9.7	L	Ga Kβ
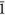 3 1	317.2	313.5	3.7	L	Ga Kβ
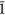 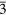 3	316.6	310.4	6.2	L	Ga Kβ
4 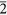 2	3.8	−6	9.8	L	Ga Kβ
2 2 0	1.3	15.2	−13.9	L	Ga Kα_1,2_
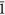 3 1	314.5	313.5	1	L	Ga Kα_1,2_
3 3 1	314	312.4	1.6	L	Ga Kα_1,2_
3 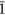 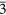	314	312.5	1.5	L	Ga Kα_1,2_
2 2 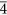	1.3	−17.5	18.8	L	Ga Kα_1,2_
3 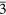 3	313.5	313.5	0	L	Ga Kα_1,2_
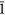 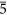 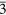	313.1	304.4	8.7	L	Ga Kα_1,2_
5 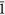 3	313.1	312.8	0.3	L	Ga Kα_1,2_
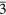 1 5	49.4	57.1	−7.7	B	Ga Kα_1,2_
4 4 4	1.3	10.5	−9.2	B	Ga Kα_1,2_
