# Supplementary material for: Experimental phase determination of the structure factor from Kossel line profile
Source: Sci Rep. 2016 Mar 11;6:22904. doi: 10.1038/srep22904 (PMC4786796; doi:10.1038/srep22904)
Supplement: Supplementary Information [file srep22904-s1.pdf]

Supplementary Information

Experimental phase determination of the structure factor from Kossel line profile

G. Faigel, G. Bortel, M. Tegze

Discussion

Design Considerations of the Experimental Setup

The sketch of our experiment is shown in Fig. 2 (article numbering). There are two critical components in the system, the focusing mirror, and the 2D pixel detector. In order to understand why these units are essential in these experiments, let us discuss the various requirements.

From the theory of dynamical diffraction we know that the fine structure of Kossel lines containing the phase information is in the arcsec range, requiring very high angular resolution. Assuming practical sample-to-detector distances, this translates to spatial resolution in the 10 μm range, limiting both the spot size of the probe beam and the pixel size of the detector.

Interference of the direct outgoing fluorescent radiation and its reflection modulates the intensity in the vicinity of the cones satisfying the Bragg condition. Theoretically, this modulation, the fine structure of a single Kossel line is on the 0 to 4 scale relative to the fluorescent radiation: for fully constructive interference it is 4, for completely destructive interference it is 0. However, in practice this contrast is much lower. The reason is mostly the non-perfect nature of the crystals, the mosaicity. This smears out the Kossel line profiles, increasing their characteristic width and decreasing the intensity modulation. This relaxes the pixel size requirement but demands for large dynamic range of the detector.

Another factor affecting the quality of the obtainable Kossel patterns is the suppression of the primary exciting x-ray radiation relative to the fluorescent x-ray radiation. This can be achieved by proper shielding, eliminating air-scattering and by using detectors with good energy separation via both lower and upper thresholds. Filters cannot be used for this purpose, as appropriate suppression of the primary radiation could be achieved only for low energy differences with unpractically thick filters causing huge intensity loss. Also the possible inhomogeneity of the filters can appear on the detected images.

It is clear from the above, that from the point of view of structure determination one should measure as large solid angle as possible, to collect many Kossel lines, while from the point of view of determining the fine structure of lines one needs high angular resolution. To obtain good contrast in the Kossel pattern one needs either as perfect crystals as possible or the detectors should have large dynamic range to handle the low contrast obtainable from non-perfect crystals.

The first two requirements lead to a huge number of pixels. Let us estimate the parameters of the detector. Today's hybrid pixel detectors have typically $50\times50\ \mu m^2$ pixels. Taking 0.001° angular resolution, the minimum distance between sample and detector is about 2 m. So, the number of pixels on a detector covering a half sphere is $\sim10^{10}$. That is absolutely unrealistic at the present level of technology. 2D detectors with $\sim10^6$ pixels are readily available. This means that we have to relax the requirements. Either we measure in a smaller solid angle, or degrade resolution, or some compromise in between.

Experimental Setup

In our setup (Fig. 2.) we used a 512×512 hybrid pixel detector, capable of direct x-ray detection. It is based on a Medipix-2 chip developed at CERN and bonded to a 300 μm thick

Si sensor [s1]. The detector was at 160 and 370 mm from the sample. Since this covers only 9°×9° and 4°×4° solid angles respectively, we extended the first range up to 9°×27° by rotating the detector about a vertical axis centered on the sample (see Fig. 2.). This setup was built at the ID18 beamline of ESRF [s2]. The primary 14.4 keV x-ray beam was monochromatized to ~1 eV bandwidth using a Si (111) channel cut monochromator. A Kirkpatrick-Baez mirror [s3] focused the beam to a 15×5 μm^2 spot on the surface of the sample. The incident intensity on the sample was $\sim 10^{14}$ photons/sec. Taking into account the fluorescent yield, geometrical factors and absorption we expect $\sim 10^4$ photons/pixel/sec in the detector. Statistically this would be enough to see the Kossel lines even if the contrast is degraded. However, in practice one cannot see them and we found, that the reason is the non-uniform response of the detector. Even after using the flat-field correction of the detector (via individual pixel threshold offsets) the statistical fluctuation is about 2 orders of magnitude larger than that corresponding to the Poisson noise (Fig. s1.). To make the patterns visible, we need custom data processing described in the next section.

Beside fluorescent radiation, x-ray photons from other processes, like Compton scattering from air and from the sample, elastic scattering from air and from the surrounding objects (like detector arms, shielding, etc.) can also reach the detector. These give an additional background, which might further degrade the contrast of the Kossel lines. Part of these can be avoided by proper shielding. Those parts, which cannot be suppressed, should be accounted for in the evaluation process. In order to measure these background contributions we installed a small solid state detector close to the 2D detector. Using this we monitored the energy distribution of the radiation reaching the 2D detector and could identify the amount of radiation originating from the various processes.

Pattern Processing

To correct for the inhomogeneity of the detector, first we applied a normalization procedure as follows: We accumulated a pattern of homogeneous or spatially slowly varying intensity distribution (normalizer pattern, *N*) under similar conditions as the pattern of the sample under study (pattern to normalize, *P*). It can be done either by spinning the sample about an axis that does not intersect the detector (to avoid a fix point) or by using an amorphous material containing the same kind of fluorescent atoms as the sample. In the case of GaAs we used the spinning method. After dividing pixel by pixel the two patterns ($K = P/N$), the inhomogeneous sensitivity pattern characteristic to the detector disappears and sharp features, such as the Kossel lines become visible. During this normalization the slowly varying component of the fluorescent background is removed that we are not interested in anyway. To further improve the visibility of the Kossel lines we made this background intensity constant by fitting a low order polynomial function to the normalized patterns (S = low frequency fit of K) and then calculated K/S, the final pattern. This brings the base fluorescent intensity to 1 and transforms the Kossel patterns to their natural intensity scale. We also did minor corrections to the patterns by replacing dead and hot pixel values by the average of their surrounding pixels. This whole procedure gives a significant improvement in the statistical properties of the patterns, but the pixel to pixel fluctuation remains one order of magnitude larger than the Poisson noise (Fig. s1.).

The next step in the evaluation is the description of the lines. For a given direct (and reciprocal) lattice, orientation matrix and fluorescent wavelength, the direction of the Kossel cones for each set of planes can be calculated. With appropriate instrumental parameters (sample to detector distance, detector size, detector scan parameters, detector tilt angles, sample displacements etc.) one can also locate the measured images in the same space. We developed a software that draws the Kossel cones on top of the preprocessed patterns and allows interactive adjustment or refinement of all structural and instrumental parameters.

Once the geometry of the experiment is perfectly described, this software is also capable to extract the Kossel line profiles for each (well and less visible) reflection by averaging measured intensities along narrow concentric cones of the given Kossel cone.

Derivation of Equations (1) and (2)

We based the calculation on the work of Hannon and coworkers [17, 18, 19] and we omitted polarization.

For the Laue case we started from equations (3) and (4) of [19]. Accordingly, the amplitude at the top layer of the sample is

$$A(\mathbf{k}) = D(\mathbf{k},\mathbf{k})J_0 + D(\mathbf{k},\mathbf{k}_1)J_1,$$

where D-s are the propagation matrices, J_0 and J_1 are the source currents. Expressing D-s with the transmission matrices T-s, the amplitude becomes

$$A(\mathbf{k}) = \frac{ik}{2\pi}\left(T(\mathbf{k},\mathbf{k})e^{-i\mathbf{k}\mathbf{r}_j}J_0 + T(\mathbf{k},\mathbf{k}_1)e^{-i\mathbf{k}_1\mathbf{r}_j}\frac{\sin\varphi_0}{\sin\varphi_1}J_1\right).$$

Next we put explicit expressions for $T(\mathbf{k},\mathbf{k})$ and $T(\mathbf{k},\mathbf{k}_1)$ using (53) and (54) of [17]

$$T(\mathbf{k},\mathbf{k}) = \left(\cos(M_j\beta) - i\nu\frac{\sin(M_j\beta)}{\sin\beta}\right)e^{iM_j(gd-\delta+\alpha)},$$

$$T(\mathbf{k},\mathbf{k}_1) = -iF^{10}\frac{\sin(M_j\beta)}{\sin\beta}e^{iM_j(gd-\delta+\alpha)-i(\delta+\alpha)}.$$

Using the facts that $\beta \ll 1$ and in our case J_0 and J_1 are the same, with a little algebra we arrive at

$$I(\delta) = |A(\mathbf{k})|^2 = B\left|\left(1+\frac{\nu}{\beta} - C\frac{F^{01}}{\beta}\right)\mathrm{e}^{\mathrm{i}M_j(\alpha+\beta)} + \left(1-\frac{\nu}{\beta} + C\frac{F^{01}}{\beta}\right)\mathrm{e}^{\mathrm{i}M_j(\alpha-\beta)}\right|^2.$$

The definition of α, β, ν, B and C is given in the main text, separately for the Laue and Bragg cases.

For the Bragg case we started from equations (31), (32), (33), (35) of [19] and (44), (47) of [17]. Again, the amplitude at the top layer of the sample

$$A(\mathbf{k}) = D(\mathbf{k},\mathbf{k})J_0 + D(\mathbf{k},\mathbf{k}_1)J_1,$$

where D-s take the explicit form

$$D(\mathbf{k},\mathbf{k}) = Q^{-1}e^{iM_j(gd-\delta+\alpha+\beta)},$$

$$D(\mathbf{k},\mathbf{k}_1) = im^*F^{01}Q^{-1}e^{iM_j(gd-\delta+\alpha+\beta)}.$$

Using the fact that $\beta \ll 1$ we arrive at

$$I(\delta) = |A(\mathbf{k})|^2 = B\left|\frac{1+\mathrm{i}m^*CF^{01}}{Q}\mathrm{e}^{\mathrm{i}M_j(\alpha+\beta+gd-\delta)}\right|^2.$$

The definition of α, β, ν, B, C, Q and m^* is given in the main text, separately for the Laue and Bragg cases.

M_j, g, d and δ are the same for both cases and they are defined in the main text.

Line-shape, Fitting, Convergence and Uniqueness

Since the line shape is strongly related to the phase of the structure factor, and the goal of this paper is to determine the phase, most of the discussion concerning the fitting of the shape was given in the main text. Here we give a few remarks, which explain some details not essential to the understanding of the main points of the paper but clarify our numerical procedure.

In the fitting procedure we included the absorption of the incident radiation, so the relative intensity reaching a layer at depth s was taken as $I_0e^{-\mu s/\sin\varphi_{\mathrm{inc}}}$, where I_0 is the intensity incident on the top surface and φ_{inc} is the angle of incidence relative to the surface of the crystal. In the case of simultaneous fitting of $K_{\alpha1}$, $K_{\alpha2}$ lines, the distance of the lines was fixed by the energy distance and Bragg angle of the proper reflection. In some cases (see for example fig. 4. Ga K_β 220 reflection) one can see an additional weak line by the main line under consideration. By carefully examining the 2D diffraction pattern, one can see that there is another non-concentric line close to the examined one. This extra line appears in a distorted

way in the averaged curve, and gives a wrong baseline. Therefore these parts are taken out of the fitting procedure.

As it is described in the main text we fitted four parameters: line position, line broadening, amplitude of the structure factor and phase of the structure factor. In ideal measuring conditions all of these parameters are uniquely determined by the sample. The composition determines line position (relative to the kinematical Bragg angle), the structure determines the amplitude and phase, and the broadening is connected to the crystal imperfections. In this experiment we concentrated to the determination of the phase. We know that by our experimental setup we cannot obtain absolute line positions and widths with a precision which would allow obtaining the physical quantities they are coupled to. Therefore we expected that these two parameters should be handled in a different way than the other two. In spite of this, in the first try of fitting we started the fits from random values of all four parameters iterating simultaneously. With this we intended to check the convergence region of the parameters. The random values were chosen from physically plausible intervals for the parameters: 0–360° for the phase; $0.1\times F_{GaAs}$–$10\times F_{GaAs}$ for the amplitude; half of the size of the full angular range for the width and the size of the full angular range for the position of the lines. We made 50 runs with different random starting parameters for two selected lines (call these 1 and 2) having the best statistics. The result was similar in the two cases: looking all 4 parameters none of the fits converged to the same (4D) point. However, examining the parameters separately we found that the position parameter was relatively stable, the amplitude and broadening were slightly correlated, while the phase in 4 (line 1) and 5 runs (line 2) converged to the same value (within 10°), meanwhile the error (mean square deviation) was the lowest in these cases. Checking the starting values of the phase in these 4 and 5 cases we found that they were within 10° distance from the mean of the fitted values. The above findings were in line with our expectation that the phase should be handled differently in the fitting procedure than the broadening and line position. Although the amplitude of the structure factor appears at the same place in the equations (1) and (2) as the phase (but in a different role) we decided to handle it not like the phase but rather like the position and the broadening parameters. The reason for this is the correlation we have seen between broadening and amplitude. The above reasoning led us to the three stage fitting process, starting with the 20° mapping of the phase and fixing the other 3 parameters to the center of the above given intervals. From the 360°/20°=18 line profiles we selected the best fit and performed the 2nd step: 3 parameter fit and phase fixed, starting from the corresponding phases. In the 3rd step we refined the phase only. We repeated the same process with map points shifted by ±5°. We found that the phases converged to the same values within 3°. This procedure gave us an estimate of the convergence of the fits and the uniqueness of the result. Since we found that this three stage process gives an unbiased, stable result we applied it for all other lines.

Experimental Considerations in XFEL Measurements

In the "*Design Considerations of the Experimental Setup*" and "*Experimental Setup*" parts we discussed synchrotron experiments. The conditions at single pulse XFEL measurements are more demanding, especially for the detector. The reason is that one has to measure in a very short time (10–100 fs) a large number of photons (10^{11}–10^{12}) in the full area of the detector. First let us see what is needed in the ideal case. As we pointed out above, the number of pixels on a detector covering a half sphere should be ~10^{10}. The number of photons in an XFEL pulse can be in the range of 10^{12}–10^{14} photons. For definiteness let us take 2×10^{13} photons/pulse and 0.1 for fluorescent yields. This choice results in 100 photons/pixel. This statistics allows the measurement of an effect larger than 10%. Assuming a crystal with mosaic spread at most 10× the theoretical line width and two fluorescent elements with two emission lines we expect the Kossel line peak intensity relative to the background fluorescent

intensity to be about 10%. The above statistical estimate satisfies this expectation. However, in this example we did not count with any other background contribution like inelastic scattering from the sample, air scattering and detector noise. Further, we assumed homogeneous and high (>90%) pixel sensitivity of the detector. At present, detectors with that much pixels and with that good characteristics do not exist. However, as our experiment shows, a relatively complete dataset (54% completeness) can be measured with a much smaller detector. Today, about 10 times larger and better quality detectors are available. For synchrotron work probably a large EIGER R or X hybrid photon counting detector (developed by Dectris) is a good choice, while for single pulse XFEL measurements an integrating detector, such as a pnCCD with a smaller size (1 Mpixels, available at LCLS) might work. However, before a real experiment one should do a more precise characterization of the pnCCD under the conditions of a Kossel pattern measurement. Note, that there is a detector development at EU-XFEL, which may result in larger and better detectors than presently available. So, using today's technical resources we expect that Kossel patterns can be collected for relatively simple samples (preferably one fluorescent element and high crystal symmetry) with >90% completeness from a single pulse. More complicated samples and more complete datasets need larger detectors and a more intense probe beam. These may be available at EU-XFEL, which start to operate in the near future.

We pointed out in the Conclusion of the main article, that the Kossel technique may facilitate the study of structural characterization of changes caused by highly non-ambient conditions. We add a few remarks here. The realization of highly non-ambient conditions is a difficult problem itself. It usually involves equipments or parts of equipments close to the sample. These parts geometrically limit probing the sample by x-ray diffraction. Further, in many cases it is difficult to keep the highly non-ambient condition for an extended period of time. Therefore, there is only a short time window for measurement. In traditional single crystal diffractometry the sample has to be rotated to many positions to collect a dataset. This prevents taking a meaningful diffractogram during a magnetic pulse or during a short pressure jump. At synchrotrons this problem is circumvented by using a white probe beam and taking a Laue pattern. However, at XFEL-s this method does not work, because XFEL is monochromatic. In this case Kossel patterns would give a lot of information on the structure. Since the sample is stationary during Kossel pattern collection, it is much easier to take into account the constraints given by the apparatus producing the non-ambient condition. This gives a good base in designing the optimum measurement strategy. From the previous paragraph it is clear that collecting a Kossel pattern using only a single XFEL pulse will be technically demanding. Adding non-ambient conditions to it will further complicate measurements. However, we believe that it is worth to put more effort to develop this technique because in those cases where we have non-repeatable conditions (which may happen often, since from the highly focused XFEL beam even a single pulse can damage the sample in an irreversible way) Kossel technique gives unique information.

References

[s1] Hatsui, T. & Graafsma, H. X-ray imaging detectors for synchrotron and XFEL sources. *IUCrJ* **2,** 371–383 (2015).

[s2] http://www.esrf.eu/UsersAndScience/Experiments/DynExtrCond/ID18

[s3] Kirkpatrick, P. & Baez, A. V. Formation of optical images by x-rays. *J. Opt. Soc. Am.* **38,** 766–774 (1948).

Figures

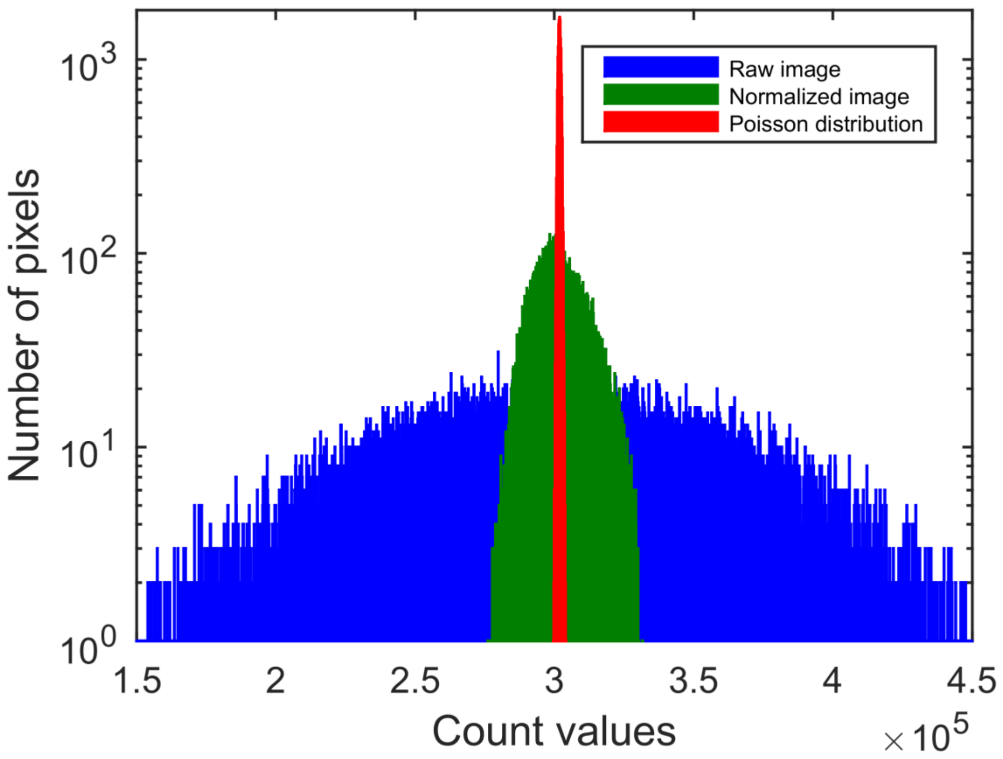

Fig. s1. Statistical properties of detector response to a homogeneous illumination. Distribution of as measured pattern (wide blue part), normalized pattern (narrower green part) and the ideal Poisson distribution (narrow red central part).

Movies

Movie 1. Phase dependence of the Kossel line profile in the symmetric Bragg case ($\varphi_s = 0°$). The crystallographic phase of the reflection is indicated by the circle in the upper right corner. The angular range is normalized to the Darwin width of the reflection, while the intensity is to the background fluorescent radiation (gray dotted lines).

Movie 2. Bragg and Laue case of Kossel lines. $\mathbf{k}_0$ and $\mathbf{k}_1$ wave vectors are located on the same side of crystal surface for the Bragg case (red), while on the opposite sides for Laue case (blue). There exist pure Bragg, pure Laue and mixed Bragg-Laue cones depending on θ_B and φ_s. Note, that $\mathbf{k}_0$ and $\mathbf{k}_1$ are wave vectors indicating the directions of the x-ray beams and not the beams themselves. The beams are in the crystal coming from the atoms as fluorescent radiation in the form of spherical waves and its scattered radiation.
